# Empirical Prediction of Turnovers in NFL Football

**DOI:** 10.3390/sports5010001

**Published:** 2016-12-29

**Authors:** Joel R. Bock

**Affiliations:** PointPredictive Inc., San Diego, CA 92120, USA; sauerkraut@gmail.com

**Keywords:** machine learning, unbalanced data, predicting rare events, NFL football, sports analytics, performance optimization

## Abstract

Turnovers in the National Football League (NFL) occur whenever a team loses possession of the ball due to a fumble, or an interception. Turnovers disrupt momentum of the offensive team, and represent lost opportunities to advance downfield and score. Teams with a positive differential turnover margin in a given game win 70% of the time. Turnovers are statistically rare events, occurring apparently randomly. These characteristics make them difficult to predict. This investigation advances the hypothesis that turnovers are predictable in NFL football. Machine learning models are developed to learn the concept: At any point within a football game, what is the likelihood that a turnover will be observed on the next play from scrimmage? NFL play-by-play data for 32 teams spanning seven full seasons were used to train the models. Results presented suggest evidence to support the working hypothesis. Under certain conditions, both fumbles and interceptions can be anticipated at low false discovery rates (less than 15%). When a turnover is predicted on the impending play from scrimmage, a high degree of confidence is associated with that prediction. The ability to anticipate catastrophic in-game events may lead to their management and control, ultimately improving the performance of individual athletes and their teams. This investigation contributes to the sports science literature by demonstrating the predictability of in-game events often considered to be essentially random in their occurrence. To the author’s knowledge, direct prediction of turnovers has not previously appeared in the literature, which has focused on retrospective statistical analyses of turnover margin in football games.

## 1. Introduction

Turnovers in the National Football League (NFL) occur whenever a team loses possession of the ball due to a fumble, or an interception. A fumble is any act other than passing, handoffs, or legally kicking the ball, which results in a loss of possession from offense to defense. Interceptions occur when a defender catches a pass or fumble that does not touch the ground [1]. Turnovers disrupt the momentum of the offensive team, and represent lost opportunities to advance downfield and score.

A positive differential turnover margin in a given game is a significant predictor correlated with winning that game [2]. One analysis of multi-season game outcomes found that teams having a single turnover less than their opponent were victorious 70% of the time [3].

Turnovers may be caused by any number of factors, including physical collisions, mistakes in play execution or lapses in player concentration. In statistical terms, turnovers are rare events. Less than 3% of passes are intercepted, and less than 1% of run plays end in fumbles being recovered by the defensive team. Turnovers appear to be random events [4]; previous work concluded that roughly equal parts chance and talent accounted for year-over-year variations in turnover differential for a given team [3]. Within a season, NFL turnovers are only weakly correlated with prior game performance [5].

These characteristics—rarity, irregular but recurrent appearance, and costliness in their effect—suggest a connection with so-called “extreme” events in dynamical systems, which are inherently difficult to predict [6].

The ability to anticipate turnovers with some degree of statistical confidence potentially offers significant value for in-game strategies to mitigate their negative consequences.

This investigation tests the hypothesis that turnovers are predictable in NFL football. Statistical models that predict the likelihood of observing a turnover on a given play from scrimmage are developed, and evaluated using play-by-play data from seven NFL seasons.

## 2. Methods

### 2.1. Gradient Boosted Machine Learning

The concept to be learned in this investigation is this: At any point within a football game, what is the likelihood that a turnover will be observed on the next play from scrimmage?

The specific objective is to learn an unknown function *F* that maps explanatory variables $x=\{x_{1},\ldots ,x_{d}\}$ to the response *y*, or F:x→y, where **x** represents the game situation, and y∈{0,1} is the binary decision (no turnover, turnover). A collection of training examples $T=\{\left(x_{i},y_{i}\right),\;i=1,\ldots N\}$ is used to estimate an approximation to *F*, or $\hat{F}\left(x\right)=y$, by an adaptive learning algorithm known as the “gradient boosting machine” [7,8].

Gradient boosting machines (GBMs) are learning algorithms that reconstruct a decision function $\hat{F}$ based on the consensus of an ensemble of classification or regression trees. New decision tree models are sequentially added to the ensemble, in order to increase the estimation accuracy of the response variable. The numerical optimization minimizes an expected loss $\hat{F}\left(x\right)={\text{argmin}}_{F}E_{y,x}L\left(y,F\left(x\right)\right)$ of a group of trees, conditioned over the entire training data set [7]. The loss function can be selected according to a given learning concept and joint probability distribution f(x,y) under study. Here, we use a Bernoulli distribution loss function, convert the classification to a continuous value via logistic regression and estimate the turnover probability $\hat{p}\left(x_{i}\right)=p\left(y_{i}=1|x_{i}\right)$, $\hat{p}\in \left[0,1\right]$ .

A useful property of GBMs in applications is interpretability through calculation of the relative influence of explanatory variables in constructing a consensus decision. The influence of each input variable $x_{j}$ in a given tree is based on the frequency of its selection for splitting in non-terminal nodes, and its contribution to successful model classification over the data sample. This influence is averaged over the ensemble of trees to estimate the variable’s overall importance to the decision function $\hat{F}$ [7]. In the current investigation, this interpretation may provide insight into the game conditions under which turnovers might be expected to occur.

Gradient boosting machine models were developed and evaluated in *R*, using the ”gbm” package [9,10].

### 2.2. Sample, Segmentation and Features

The population under study consists of NFL season, game, player and play-level data for complete seasons 2009 through 2015, covering all 32 teams. Game data were downloaded from the site www.nfl.com using utilities provided by ”nflscrapR” [11]. These data were preprocessed by (1) sampling by season and team; (2) filtering by play type, to include only plays from scrimmage (run, pass or sack); (3) annotating by status of turnovers (true, false) observed on each play; (4) constructing feature vectors using attributes of the play-by-play and game contextual data.

This sample comprised 300,450 plays. Running plays represented 31.7% of all plays, passes 42.1%, and sacks only 2.9%. Although sack-fumbles lost are significant events (5.1% of sacks produce turnovers), we decided to exclude sacks from further consideration due to their negligible numbers relative to run and pass plays. To make this predictive analysis useful in practice, it is prudent to categorize turnover events in association with scrimmage plays that could reasonably be anticipated by a defensive team, based on offensive formation.

After excluding sack plays, the sample contained 291,675 plays, with an overall turnover prevalence of 1.633% for pass and run plays, combined. Pass plays made up 43.4% and runs 32.6% of the resultant dataset.

Two partitioning schemes were applied to the sample. First, an aggregate sample of all 32 NFL teams was created to assess whether invariant patterns of turnover predictability could be determined. Second, individual team samples were assembled to develop team-specific models of turnovers. Seven full season-long records were used for all sample datasets.

Predictive models were trained and evaluated for each sample. These samples were segmented by distinct event types—(1) *Run* plays; (2) *Pass* plays; and (3) *Run or Pass* plays combined.

Feature vectors for learning were constructed from available fields in the play-by-play data. Numeric data were normalized by characteristic length and time scales. Categorical and ordinal variables were represented as binary valued quantities using one-hot encoding. The features and their corresponding nominal dimensions upon encoding are summarized in Table 1. Not all dimensions listed in the table were in model development, due to their low variation across certain limited subsamples.

### 2.3. Modeling and Analysis

The incidence of turnovers as a percentage of all plays from scrimmage is very low, around 1.6%. For this reason, the distribution of class labels $y_{i}$ in a training set $T=\{\left(x_{i},y_{i}\right),\;i=1,\ldots N\}$ randomly sampled from the true population is highly skewed. Learning the parameters of a useful statistical estimator of turnover probability $\hat{p}\left(x_{i}\right)=p\left(y_{i}=1|x_{i}\right)$ suggests the use of specific learning techniques to avoid trivially predicting “no turnover” on every decision [12].

To address this, the approach taken in this study was to re-balance the distribution of classes in the training set, over-representing the distribution of the minority class in order to present sufficient examples to the learning algorithm. During validation of the models, examples closely representative of the true distribution within the population were used to assess model predictive power when applied out-of-sample.

The modeling strategy included bootstrap resampling [13], cross validation analysis, and receiver operating characteristic curve (ROC) analysis [14]. The latter technique enabled error estimation, model comparison and selection from the large number of hypotheses generated by the gradient boosting machines during training. ROC curves are often used to tradeoff false positive rate (FPR) and true positive rate (TPR) for evaluation of classifiers. In this study, the false discovery rate (FDR) was substituted for FPR for analysis. FDR is the fraction of all positive decisions (i.e., turnover predicted) made by a model that are incorrect [15]. FDR is a more informative metric than FPR in diagnostic or predictive applications where confidence in a positive prediction is preferred, especially when the class distribution is skewed [16]. FDR is related to the positive predictive value statistic by PPV=1-FDR. High PPV (low FDR) values are desirable. TPR denotes the sensitivity of the model, or the likelihood that actual turnovers events are detected within a testing distribution.

In ROC space (FDR,TPR), an optimal decision threshold $DT_{opt}$ is determined experimentally for a given distribution. Our objective is to minimize FDR for tactical reasons. A second pass through the training data with this fixed threshold is used to train and evaluate model performance. The gradient boosted model outputs a probability $\hat{p}$; the turnover prediction algorithm is then [17]

$$\hat{y}\left(x\right)=\left\{\begin{array}{cc} 1: & \;\mathrm{if}\;\hat{p}>DT_{opt}\\ 0: & \;\mathrm{if}\;\hat{p}\leq DT_{opt} \end{array}\right.$$

where $\hat{y}\left(x\right)=1$ means a turnover will be observed given input x.

Model learning for the aggregate sample used the “bootstrap” [13] to repeatedly draw samples from the entire training set. Data were partitioned according to the play type segment under consideration, and a stratified sample was constructed for training. The validation data were sampled at random from the entire sample, according to the natural distribution of turnovers. A two step procedure was followed, for each of B=100 bootstrap replicates. The first step estimated the detection threshold (DT) for optimal FDR and TPR via ROC analysis, training GBMs comprising 1500 trees (nominally). Secondly, the threshold was held constant such that $DT=DT_{opt}$ and the entire sample was modeled again.

The learning procedure used for the team-wise samples was notionally similar, differing slightly in the numerical mechanics. Stratified sampling (with respect to the class labels *y*) of individual teams produced untenably small sample counts. This required an alternate sampling strategy. The decision was made to use 10-fold cross validation, nested within a 10-trial bagging procedure. Prediction rules were developed by finally averaging the performance results. Modeling therefore included all of the available instances, and benefited from the variance-reduction properties of bagging for model performance estimation [18].

Performance statistics $FDR\left(DT_{opt}\right),TPR\left(DT_{opt}\right)$ were accumulated and finally averaged over replicates *B* (or trials/folds *k*) to estimate the generalization performance of the ensemble of trees. Sampling distributions of the sample mean and standard error values for FDR and TPR observed in out-of-sample test were recorded for each sample and segment under investigation.

In this investigation, we define a “good” false discovery rate to be FDR<0.15. In other words, a positive prediction made by the model ($\hat{y}\left(x\right)=1$) is correct at least 85% of the time to meet this criterion of model utility. This means that when a turnover is predicted on the impending play from scrimmage, a high degree of confidence can be associated with that prediction.

A pseudo-code outline of the model training and evaluation procedure appears in the Appendix, as Algorithm A1.

## 3. Results

The main predictive modeling results of this paper are presented in Table 2, Table 3, Table 4 and Table 5. These tables list the sampling distributions of the sample mean and standard errors for observed false discovery (FDR) and true positive rates (TPR) on predicting turnovers for the sample data segments and event types considered. These are the primary statistics used to evaluate turnover prediction acuity. The results tables also include observed values for prevalence of actual turnovers (*Prev.*) and corresponding out-of-sample instance counts ($N_{oos}$).

We define a “good” false discovery rate to be FDR<0.15. Tabulated results are annotated in green to highlight predictions meeting or exceeding this threshold for low FDR. True positive rates observed, where TPR>0.60, are similarly noted in the tables.

Team-aggregated performance results are found in Table 2. Model predictions by individual team and segment appear in Table 3, Table 4 and Table 5 .

A synopsis of the important input variables contributing to the ensemble prediction for one of the samples studied is presented in Table 6.

### 3.1. Turnover Prediction Results—Aggregated

Table 2 summarizes performance of models for the aggregate sample, for each of the three turnover event types considered. Low values of FDR are seen for each segment (6.4%,8.3%, and 3.9% for *Run or Pass*, *Pass* and *Run*, respectively). For Run plays, the sensitivity TPR is seen to be 0.65, making this segment the most successfully predicted turnover event type in the sample.

The results shown in Table 2 lend support to our hypothesis that turnovers are predictable in NFL football. Given an educated guess as to the expected play type from scrimmage, the GBM models have excellent positive predictive value in estimating turnover likelihood.

### 3.2. Turnover Prediction Results—Team-Wise

Prediction results for team-wise samples of the *Run or Pass* segment are found in Table 3. These statistics show that 20 of the 32 team models (62.5%) are observed to have FDR rates below the 0.15 goodness criterion as defined here. The best performing team model in terms of false discovery rate was for the New England Patriots (NE), where the sample mean FDR was 2.1%. Cleveland (CLE), Buffalo (BUF), San Diego (SD), Minnesota (MIN), Tennessee (TEN) and Seattle (SEA) each had FDR’s of less than 10%. San Francisco (SF) produced the most frequent rate of false positives, at 26.9%.

Note that the standard errors of the sample mean of the FDR statistic are a significant proportion of the sample mean value. This behavior is seen in nearly all of the turnover prediction results obtained in this study. Model sensitivity TPR is moderate in general, ranging from 40% to 50%. This statistic is considered less vital here than FDR, but is still important.

Details of modeling performance for *Pass* plays from scrimmage are presented in Table 4. For this segment, 10 of 32 teams (32%) were associated with good turnover predictability as measured by FDR. The best precision was observed for Tennessee, where FDR=0.077. New England had the highest false positive rate at 30%. The true positive rate of the method is seen to be generally low, in the 30% to low 40% range.

Large standard errors of the predicted sample mean FDR are apparent in these results.

Statistical performance of models trained on *Run* segment data is compiled in Table 5. These models are seen to be less precise than observed for other segments, as 7 of 32 team models produced sample mean FDR’s of less than 15%. It is clear that the overall sensitivity of the *Run* turnover predictions is very good, as 69% of the models (22/32) show true positive rates exceeding 60%. This observation is consistent with the “rolled-up” results shown in Table 2, where the overall *Run* segment produces TPR=0.65.

It is interesting to note that the lowest turnover prevalence by play type in the population (for run plays) is associated with the highest degree of model sensitivity in out-of-sample predictions. The disparity in realized false positive rates between Table 2 and Table 5 could be, in part, explained by the absolute number of training observations available in the aggregate case versus the team-wise samples, where fewer examples in the latter may be insufficient to learn the joint distribution between input and output variables.

### 3.3. Variable Influence

The relative importance of variables in the feature vector is estimated by gradient boosted machines [7]. This estimate is based on the increase in log likelihood of a decision tree as determined by non-terminal splits made on each variable; this likelihood improvement is summed over all trees in the ensemble to apportion that variable’s contribution to the plurality decision.

The most and least influential variables for turnover predictions made for the aggregate sample are summarized in Table 6. The most important features are last_pass_complete and last_pass_incomplete. These are boolean valued features describing the outcome of the last pass from scrimmage. The remaining highly influential variables make intuitive sense, describing the current game situation (time remaining, score differential, yards to go for a first down or score). Two seemingly trivial variables (is_pass and is_run) are consistent with the composition of the *Run or Pass* segmentation, and strongly influence the splitting pattern of the constituent trees.

Non-important variables include the quarter of the game (subsumed by time_left), the direction of a current run play, and plays immediately following special teams activity.

## 4. Discussion

The hypothesis motivating this study was that turnovers in NFL professional football are predictable. Statistical models were trained to predict the likelihood of observing a turnover on a given play from scrimmage. Empirical data representing seven complete NFL seasons (2009–2015) were used to train, test and evaluate the models.

Our machine learning results suggest evidence to support the hypothesis. Under certain conditions, both fumbles and interceptions can be anticipated at low false discovery rates (less than 15%). This means that when a turnover is predicted on the impending play from scrimmage, a high degree of confidence can be associated with that prediction.

The operational premise from the defender’s perspective is that the impending play type (*Run*, *Pass*, *Run or Pass*) can be reliably estimated, using statistics (e.g., [19]) or intuition, in advance of the ball snap. These three play type categories are the basis for data segmentation, and predictive models are developed for each in turn.

For coaching purposes, this approach may be useful during development of game plans, or to inform in-game strategies to mitigate the negative consequences of turnovers by an offensive team, or to maximize their probability by a defensive squad.

Turnovers occur for many reasons, both physical and mental. Interceptions are caused by many factors—tipped balls and errant passes miss their destination due to the pressure of a defensive pass rush; misreading the defensive scheme prior to the ball snap; excellent coverage downfield by defensive backs; lack of spatial awareness of players on the field. Fumbles can be produced by violent physical collisions; insufficient ball protection by the runner; defensive “stripping” of the ball during tackling; or quarterback sacks resulting in fumbles.

This investigation contributes to the sports science literature by demonstrating the predictability of in-game events often considered to be essentially random in their occurrence. To the author’s knowledge, direct prediction of turnovers has not previously appeared in the literature, which has focused on retrospective statistical analyses of turnover margin in football games.

### 4.1. Quantitative Results and Observations

Two data samples were modeled to assess turnover predictability: an aggregate of all NFL teams, and 32 individual team samples. Seven full season-long records were used for all datasets. We define a “good” false discovery rate to be FDR<0.15. A positive prediction made by a model ($\hat{y}\left(x\right)=1$) is correct at least 85% of the time.

The GBM models to predict turnovers generally are characterized by high positive predictive value, and moderate sensitivity.

Predictive results for the aggregated sample (Table 2) exhibit low false discovery rates (FDR) for each play type segment (6.4%,8.3%, and 3.9% for *Run or Pass*, *Pass* and *Run*, respectively). These measures reflect outstanding positive predictive value in estimating turnover likelihood. Run plays displayed the greatest sensitivity, with TPR=0.65%.

Team-wise turnover predictions are summarized in Table 3, Table 4 and Table 5.

For the *Run or Pass* play segment, three-fifths of the team models (62.5%) have FDR rates below the stated 15% goodness criterion. Sensitivities were moderate for all team models. This exemplifies the essential tradeoff between FDR and TPR in ROC space [14], and follows from the present objective of minimizing FDR for strategic utility of the method.

*Pass* play results showed that one-third of team models (32%) had false discovery rates below the goodness threshold. TPR is in the 30%–40% range.

For *Run* plays, 22% of the team results displayed FDR<0.15. Sensitivity of the turnover predictions is good, as 69% of the models (22/32) had true positive rates greater than 60%.

It is interesting to note that the lowest turnover prevalence by play type in the population (for *Run* plays) is associated with the highest degree of model sensitivity in out-of-sample predictions. The disparity in realized false positive rates between Table 2 and Table 5 could, in part, be due to the absolute number of training observations available in the aggregate case versus the team-wise samples. Fewer examples in the latter may be insufficient to learn the joint distribution between input and output variables.

### 4.2. Extreme Events Modeling and Sports

Turnovers have a number of characteristics in common with “extreme” events produced by complex dynamical systems, which are inherently difficult to predict [6]. These attributes include the following:

(a) *Low occurrence frequency*. Previous estimates found an average 2.9% of passes by NFL quarterbacks were intercepted [4]; fumbles were turned over to the opposing team in 0.83% of all run and pass plays [20]. The present study covering years 2009–2015 shows average turnover rates of 2.4% and 0.6% for pass and run plays, respectively.
(b) *Intermittency*. Turnovers appear to be random events. In one study, nearly equal parts luck and talent were proposed to account to year-over-year variations in turnover differential for a given team [3]. Within a season, NFL turnovers correlate weakly with prior game performance . A team with a strong season-to-date record of winning the turnover battle is likely to regress to the mean; conversely, teams losing in turnover margin at a point within a season tend to improve on this statistic moving forward [5].
(c) *Costliness*. A positive turnover margin in a given game is a significant predictor correlated with winning that game [2]. Teams with a unit valued positive turnover margin with respect to the opponent win the game 70% of the time [3].

The methods developed here are informed by the nonlinear geophysical dynamics literature by framing the prediction as a classification task (e.g., [21]), using game state and its recent history as precursors to learn the turnover event. A related investigation from the sports scientific literature suggested the predictability of within-game events and associated outcomes using dynamical process modeling [22]. In that study, researchers found patterns of tempo and in-game scoring that were common to many different sports (American football, hockey, and basketball). They suggest that these cross-cutting themes may provide insight into psychological processes that affect teams’ performance in a general manner.

### 4.3. Standard Errors of Predictions

The statistics used to evaluate predictive performance reflect the sampling distribution of sample means and their standard errors, averaged over numerous replicates, folds and trials. It is observed that the standard errors for FDR are a significant fraction of the sample mean value. This holds true for most results obtained in this study. The standard error of the sample mean will decrease as $1/\sqrt{n}$, where *n* is the sample size, according to the Central Limit Theorem.

### 4.4. Sampling Notes

This study centered on macro-level game data, to test the hypothesis “turnovers are predictable” in NFL football. Our samples aggregated over teams, and over players for team-level predictive models. In extensions to this work, player-level data could be included to build individual player models; such models may provide additional insights beyond descriptive statistical summaries of turnovers in current usage.

Turnovers related to sacks were excluded from consideration, mainly due to their negligible sample size relative to run and pass plays. Only 5.1% of sacks produce turnovers.

## Figures and Tables

**Table 1:** Features used in models to predict National Football League (NFL) turnovers.

Feature	Dimension
Drive number	1
Quarter	5
Down	4
Remaining game time	1
Yard line	1
Yards to 1st down	1
Yards on drive, net	1
Play type	14
Score differential	1
Run location	3
Pass location	3
Last run location	3
Last pass location	3
Last pass outcome	3
Last play yards gained	3

**Table 2:** Aggregate prediction performance: false discovery rate (FDR) and true positive rate (TPR) of models. A total of 32 teams, seasons 2009–2015. B=100.

Segment	FDR (±se)	TPR (±se)	Prev.	$N_{\mathrm{oos}}^{B}$
*Run or Pass*	0.064 (0.026)	0.417 (0.023)	0.0164	22,183
*Pass*	0.083 (0.031)	0.381 (0.028)	0.024	12,653
*Run*	0.039 (0.035)	0.650 (0.064)	0.006	9514

**Table 3:** Team-wise prediction performance, *Run or Pass* segment, seasons 2009–2015. k=10.

Team	FDR (±se)	TPR (±se)	Prev.	$N_{\mathrm{oos}}^{k}$
NE	0.021 (0.073)	0.449 (0.112)	0.008	6545
CLE	0.087 (0.109)	0.467 (0.133)	0.018	6117
CHI	0.134 (0.124)	0.359 (0.125)	0.019	5974
HOU	0.177 (0.168)	0.453 (0.138)	0.014	6475
NYJ	0.193 (0.162)	0.431 (0.105)	0.019	6378
BUF	0.097 (0.097)	0.461 (0.151)	0.020	6072
WAS	0.117 (0.138)	0.420 (0.097)	0.017	6161
JAC	0.132 (0.116)	0.429 (0.118)	0.018	6042
STL	0.146 (0.110)	0.531 (0.108)	0.016	5527
ARI	0.135 (0.119)	0.408 (0.120)	0.020	6081
SD	0.063 (0.111)	0.493 (0.074)	0.014	6222
TB	0.164 (0.124)	0.472 (0.113)	0.019	5940
OAK	0.113 (0.120)	0.453 (0.146)	0.019	6112
DEN	0.174 (0.126)	0.491 (0.143)	0.015	6454
DAL	0.180 (0.151)	0.412 (0.112)	0.015	6108
ATL	0.177 (0.171)	0.433 (0.139)	0.015	6403
SF	0.269 (0.194)	0.446 (0.208)	0.010	5802
KC	0.195 (0.194)	0.539 (0.203)	0.014	6073
CAR	0.189 (0.147)	0.425 (0.134)	0.016	6164
PIT	0.116 (0.124)	0.498 (0.125)	0.016	6111
NO	0.189 (0.208)	0.463 (0.141)	0.015	6584
MIN	0.068 (0.110)	0.451 (0.086)	0.016	6031
CIN	0.128 (0.113)	0.507 (0.141)	0.018	6272
NYG	0.103 (0.092)	0.480 (0.147)	0.021	6291
PHI	0.124 (0.110)	0.478 (0.131)	0.019	6398
GB	0.188 (0.185)	0.485 (0.176)	0.010	6142
IND	0.140 (0.131)	0.431 (0.125)	0.017	6360
TEN	0.091 (0.102)	0.473 (0.112)	0.020	5876
BAL	0.126 (0.116)	0.531 (0.102)	0.015	6321
MIA	0.113 (0.126)	0.493 (0.121)	0.017	6089
SEA	0.097 (0.171)	0.551 (0.191)	0.013	6000
DET	0.113 (0.121)	0.452 (0.075)	0.019	6574

Team abbreviations: NE: New England Patriots; CLE: Cleveland Browns; CHI: Chicago Bears; HOU: Houston Texans; NYJ: New York Jets; BUF: Buffalo Bills; WAS: Washington Redskins; JAC: Jacksonville Jaguars; STL: St. Louis Rams; ARI: Arizona Cardinals; SD: San Diego Chargers; TB: Tampa Bay Buccaneers; OAK: Oakland Raiders; DEN: Denver Broncos; DAL: Dallas Cowboys; ATL: Atlanta Falcons; SF: San Francisco 49ers; KC: Kansas City Chiefs; CAR: Carolina Panthers; PIT: Pittsburgh Steelers; NO: New Orleans Saints; MIN: Minnesota Vikings; CIN: Cincinnati Bengals; NYG: New York Giants; PHI: Philadelphia Eagles; GB: Green Bay Packers; IND: Indianapolis Colts; TEN: Tennessee Titans; BAL: Baltimore Ravens; MIA: Miami Dolphins; SEA: Seattle Seahawks; DET: Detroit Lions.

**Table 4:** Team-wise prediction performance, *Pass* segment, seasons 2009–2015. k=10.

Team	FDR (±se)	TPR (±se)	Prev.	$N_{\mathrm{oos}}^{k}$
NE	0.305 (0.222)	0.469 (0.168)	0.011	3807
CLE	0.119 0.115)	0.445 (0.146)	0.026	3527
CHI	0.191 (0.147)	0.385 (0.134)	0.030	3404
HOU	0.174 (0.183)	0.376 (0.155)	0.020	3558
NYJ	0.205 (0.170)	0.339 (0.109)	0.032	3254
BUF	0.169 (0.146)	0.427 (0.176)	0.032	3327
WAS	0.229 (0.162)	0.415 (0.101)	0.026	3561
JAC	0.156 (0.134)	0.409 (0.138)	0.027	3485
STL	0.183 (0.189)	0.430 (0.165)	0.022	3400
ARI	0.134 (0.137)	0.400 (0.134)	0.028	3682
SD	0.120 (0.159)	0.447 (0.097)	0.021	3605
TB	0.204 (0.154)	0.449 (0.129)	0.027	3434
OAK	0.184 (0.168)	0.429 (0.143)	0.027	3535
DEN	0.149 (0.180)	0.428 (0.177)	0.022	3685
DAL	0.177 (0.161)	0.411 (0.111)	0.024	3603
ATL	0.176 (0.173)	0.434 (0.131)	0.021	3877
SF	0.269 (0.288)	0.345 (0.222)	0.016	3035
KC	0.196 (0.208)	0.433 (0.241	0.021	3183
CAR	0.246 (0.232)	0.377 (0.151)	0.026	3172
PIT	0.137 (0.139)	0.384 (0.130)	0.022	3545
NO	0.179 (0.192)	0.431 (0.157)	0.021	4119
MIN	0.198 (0.182)	0.420 (0.109)	0.025	3269
CIN	0.116 (0.122)	0.439 (0.150)	0.029	3427
NYG	0.164 (0.153)	0.378 (0.151)	0.029	3679
PHI	0.178 (0.138)	0.415 (0.117)	0.027	3698
GB	0.129 (0.161)	0.507 (0.190)	0.014	3504
IND	0.187 (0.156)	0.395 (0.118)	0.024	3936
TEN	0.077 (0.099)	0.455 (0.104)	0.029	3362
BAL	0.162 (0.150)	0.480 (0.104)	0.021	3599
MIA	0.150 (0.175)	0.408 (0.149)	0.024	3510
SEA	0.146 (0.204)	0.540 (0.206)	0.021	3127
DET	0.118 (0.116)	0.393 (0.080)	0.026	4133

**Table 5:** Team-wise prediction performance, *Run* segment, seasons 2009–2015. k=10.

Team	FDR (±se)	TPR (±se)	Prev.	$N_{\mathrm{oos}}^{k}$
NE	0.945 (0.151)	0.119 (0.321)	0.003	2537
CLE	0.230 (0.325)	0.657 (0.429)	0.006	2589
CHI	0.918 (0.195)	0.181 (0.375)	0.004	2569
HOU	0.196 (0.336)	0.762 (0.362)	0.006	2917
NYJ	0.119 (0.209)	0.778 (0.441)	0.006	2895
BUF	0.500 (0.343)	0.683 (0.404)	0.006	2744
WAS	0.335 (0.454)	0.236 (0.319)	0.005	2600
JAC	0.777 (0.287)	0.463 (0.442)	0.005	2557
STL	0.106 (0.195)	0.901 (0.161)	0.007	2565
ARI	0.571 (0.362)	0.591 (0.378)	0.008	2351
SD	0.143 (0.339)	0.665 (0.344)	0.005	2617
TB	0.317 (0.373)	0.660 (0.400)	0.008	2506
OAK	0.306 (0.353)	0.668 (0.394)	0.007	2577
DEN	0.389 (0.332)	0.603 (0.362)	0.007	2769
DAL	0.107 (0.289)	0.138 (0.328)	0.004	2505
ATL	0.337 (0.353)	0.293 (0.230)	0.005	2527
SF	0.550 (0.415)	0.728 (0.405)	0.004	2721
KC	0.279 (0.370)	0.827 (0.248)	0.007	2890
CAR	0.266 (0.278)	0.707 (0.326)	0.005	2943
PIT	0.285 (0.348)	0.913 (0.219)	0.008	2566
NO	0.937 (0.111)	0.321 (0.417)	0.004	2465
MIN	0.182 (0.274)	0.733 (0.244)	0.006	2761
CIN	0.085 (0.170)	0.770 (0.329)	0.006	2844
NYG	0.131 (0.185)	0.867 (0.201)	0.010	2611
PHI	0.357 (0.326)	0.642 (0.433)	0.009	2700
GB	0.981 (0.055)	0.119 (0.267)	0.004	2638
IND	0.224 (0.389)	0.711 (0.392)	0.007	2424
TEN	0.155 (0.218)	0.650 (0.255)	0.009	2514
BAL	0.177 (0.266)	0.858 (0.271)	0.006	2722
MIA	0.274 (0.318)	0.807 (0.331)	0.007	2578
SEA	0.658 (0.365)	0.302 (0.400)	0.004	2874
DET	0.126 (0.202)	0.750 (0.253)	0.008	2441

**Table 6:** Most and least influential variables. Aggregate sample, *Run or Pass* segment.

(a) Most Influential		(b) Least Influential	
**Variable**	**Avg. Rank**	**Variable**	**Avg. Rank**
last_pass_complete	1	qtr2	27
last_pass_incomplete	2	qtr4	28
is_last_pass	3	is_last_punt	29
time_left	4	is_run_left	30
last_yards_gained	5	qtr1	31
dwn1	6	is_run_right	32
yds_drive	7	is_run_middle	33
is_run	8	is_last_ex_pt	34
is_pass	9	is_ot	35
score_diff	10	is_last_fld_goal	36
yds_togo	11	is_last_qb_kneel	37
yrds100	12	is_last_os_kick	38

## Appendix A. Model Training Algorithms

The algorithm used to train and evaluate GBM models for NFL turnover prediction is sketched in the pseudo-code listing in Algorithm A1. This procedure was used for the aggregate sample (all teams, seasons 2009–2015). The scheme used for the teamwise samples is notionally similar. Details are described in Section 2.3.

**Algorithm A1** Pseudo-code for aggregate modeling procedure.
Z←load_data() B←100 V←0.10 F←3.0 seg←select_one(Both,Pass,Run) ntree←1500 DT←0.97 Perf[:,B]←0 **for** b←1,B **do** [Z_oos,Z_mdl]←partition(Z) o←sample(Z,V∗nrow(Z),seg) Z_oos←Z[+o,:] Z_mdl←Z[-o,:] s<-strat_sample(Z_mdl,F,seg) Z_mdl←Z_mdl[s,:] mdl←gbm(Z_mdl(ntree,…)) $\hat{y}\leftarrow predict\left(mdl,Z\_oos\right)$ $\left[FDR,TPR\right]\leftarrow roc\left(\hat{y},y,DT\right)$ Perf[:,b]←accum(FDR,TPR,…) **end for**
